# Mercurial Treatment of Syphilis

**Published:** 1878-05

**Authors:** 


### Mercurial Treatment Syphilis.

Fifty years ago no one disputed the propriety, if not necessity, of mercury in the treatment of secondary syphilis. The war against it commenced, in our country, with the crusade against regular medicine instituted by the ignorant but shrewd Samuel Thomson, who succeeded in inoculating the vulgar crowd with his own rank prejudices against " mercurial medicines, and out of whose chaotic system has grown the " Eclectic " sect of the present day. About the same time the more refined and transcendental notions of Hahneman began to pervade the higher ranks of society, and these two influences, co-operating with the acknowledged abuse of mercury by regular practitioners, led to its extensive abandomnent by the latter. Professional men began to treat syphilis without mercury, often with the motive, not altogether professional, of catering to public prejudice. In this they were aided by the introduction of certain new preparations of iodine. Finally there grew up almost an entire generation of physicians imbued with a prejudice against mercury, and refusing to employ it in syphilitic disorders. By-and-by the current began to turn. The non-mercurial treatment began to fail. Its cures were found to be only temporary. Within the last twenty years the profession has gradually been reverting to the old treatment. And although potassic iodide is still universally regarded as invaluable in the earlier stages, yet there is scarcely to be found at the present time, in America or in Europe, one prominent authority in medicine on the side of the exclusive non-mercurial treatment. These reflections were suggested by reading in " The Doctor " the report of a discussion in the Medical Society of London, in which Dr. Drysdale, who had been known as a distinguished opponent of the mercurial practice, declared that he had resumed it. He employed it in conjunction with large doses of iodide of potassium—15 grains. Dr. Fothergill congratulated him on his resumption of mercury. His practice was to combine the per-chloride with the tincture of iron, and since he used the combination he had had no cases of salivation. Dr. Brudenell Carter said the cure by iodide was not permanent. A relapse will occur in six months. In secondary syphilis he continued the murcurial treatment for nine months. Mr. Bloxam looked on a course of iodide as a preparation for mercury. He continued the latter twelve or eighteen months. In the Lock Hospital, preference was given to inunction, used conjointly with blue pill and opium. The iodides of mercury cause diarrhœa and salivation.—*Pacific Med. and Surg. Jovr.*

